# New York Odontological Society

**Published:** 1877-02

**Authors:** 


### ARTICLE IV.

### *New York Odontological Society*—Continued.

Dr. W. H. Atkinson. I should be sorry to have that paper go before pupils who have powers to develop. The writer said that it was the intelligence that was behind the instrument that made the instrument of value. Keep that in mind. The difficulty with those persons who are opposed to the instrument is, that they have not learned how to handle it. They have not been able to send that kindly intent that the doctor speaks of through to the object.

The paper says a wheel can go so rapidly as to wind the water around it like a skein of yarn, and the steamboat stand still. I no not know anything of that kind. I could conceive of the shafts being broken, or the buckets being riddled out of the wheel, and the wheel having no effect upon the boat; but I cannot see in any such thing as that the authority to pronounce a charge of well-settled natural laws. How can he apply that to the motion of the engine, and state that it defeats its own object? Whenever we have a cutting instrument,—and what is the bur but a repetition of excavators ?—so many fine edges,—and revolve it rapidly, more rapidly than the waves of sensation can be possibly transmitted by the nerves; in place of defeating your object, you accomplish it so quickly that it seems as though, to both patient and operator, it had hardly begun.

We would better be careful about pronouncing our notions of philosophy as final, unless we have seen all that enters into the principles that we are dealing with. If you can accustom the carrying of the weight, as he states the barber carried the weight of the razor in the hand, then you forget that entirely, and give attention only to the slight vibration of the instrument in motion; and this is possible, because I have experienced it even with the heavy Green engine. At first the weight made my hand ache, but after a while I learned so as to relieve my hand entirely.

Another fallacy: that an instrument with a spring can cut more delicately and certainly than one without. Whenever you use the spring instrument you have added ambiguity to the result of the operation, and a man must be more than a delicate operator to regulate the effect of the spring and, whoever uses it does not seem to feel satisfied until he has felt that spring. Suppose that you are within one scant layer of exposing a pulp; the spring may be just enough to pop into the chamber, no matter how careful; and we know we may pop into the chamber with any instrument if careless.

Dr. W. H. Dwinelle. I was unfortunate in not hearing the whole of Dr. Atkinson's speech. I can only speak in reference to the few observations he made subsequent to my return into the room.

I was mistaken in using the figure of a skein of silk; it does not apply. I ought to have said that a very high speed would be ineffective, because by its rapid motion the water was rejected and passed over by the paddle-wheels, they not laying hold on it. It loses its *bite* on the water.

We have all seen the driving-wheel of a locomotive rotate rapidly on the track without moving itself at all; sometimes it is necessary to scatter sand on the track before it will take a *bite* and proceed. Ice in the winter and Western grasshoppers in the summer stop our railroad trains, because the greased or frosty track will not permit the driving-wheel to bite to, or adhere to, the track. The idea of bite or no bite is not so silly after all; the principle is recognized in mechanics every day throughout the world.

I did not intend, strictly speaking, to intimate that the steamboat would stand still if you put too much power into it. I meant relatively. I meant that there is a point beyond which the buckets of the paddle wheel do not have a legitimate effect upon the water, and you lose power to no purpose. It is, so to speak, without a bite. It does not take hold on the water as it does with the limited rotations. I think I am established in this proposition. We do know that you can give such a rotation to an instrument like a bur or a drill that it will not take hold. That is a principle that is recognized by mechanics. It is a well-known fact that in cutting with machinery in iron turnings and work of that character, it is essential that rotation should be slow. You go beyond a limited rotation and you lose your bite. It is a notorious fact, and we can verify it every day in our practice with the burring engine. Take the best-formed, most acute, and best-tempered drill, set it going with this very rapid motion, and it is ineffective, comparatively speaking; reduce the motion and a few revolutions will be equal to thousands. I know that it is a fact that with a rotary lance, in the form of a wheel, you can give such a degree of motion that it will pass through flesh unperceived. I adopt the mode of removing the tool, and replacing it, as it thus frees itself from *debris* and is prevented from heating.

Dr. Atkinson. Can it heat without taking a bite?

Dr. Dwinelle. I think it can. A paper was read lately, by one of our members, in reference to the burring engine. The author informed us how rapidly he operated; how many teeth he could fill in an hour. He averaged one in about fifteen minutes. I think, as a rule, rapid operations are not good operations. You cannot do work requiring study and delicate and thorough manipulation and do it rapidly. It is true, as Dr. Atkinson said upon that occasion, we can, by the facilities we enjoy, perform many operations rapidly and thoroughly; but we cannot average our three, four, or five stoppings per hour without having it imputed to us that we are either inspired or else there is something rotten in Denmark.

Dr. A. H. Brockway. I had no idea when the paper was read that it would provoke discussion, and not until the remarks that have just been made did I suppose that I was called upon to say anything in defence of it. I thought, when I heard Dr. Dwinelle's paper, that I almost quite agreed with him. I saw very little to take exception to; and I think now that we generally agree. The expression, "rapid operating," is a comparative one. The criticisms which Dr. Dwinelle applies to me would apply to him with equal force by slower operators than himself. I differ from him in no degree except that I possibly work faster to attain the same results. He would advocate doing the thing as rapidly as is consistent with the degree of success at which he aims. I do no more. He would advocate sparing his patient as much time and suffering as possible. I do nothing different. I simply claim that with the burring engine, properly handled, and the rubber dam, I can do operations to my satisfaction three or four times as rapidly as without them. That is the pith of the matter. Whether I do them well or not is not for me to say.

If any one supposes that I said in my paper that I averaged fifteen minutes for filling cavities with gold, he is mistaken. I simply said that my average, for a certain number of days, was slightly less than fifteen minutes per cavity. I do not think that I average over fifteen minutes per cavity, taking gold, amalgam, Hill's stopping, oxychloride of zinc, or tin-foil. I did not say fifteen minutes per filling, but per cavity. Sometimes I fill two cavities, as I did to-day, in a second upper molar, at one operation. This I call two fillings. What proportion are gold fillings I am not able to say. I should say not less than one-half.

Dr. Dwinelle. Are not most of the remainder Hill's stopping?

Dr. Brockway. No, sir. I do not know when I have used Hill's stopping. Perhaps in that particular time I may have used it in a number of cases. I frequently use it for children, and as a temporary stopping in adults.

Dr. Dwinelle. I never heard of a man before who performed so many operations in an hour and did them thoroughly, and I congratulate the doctor upon his remarkable success.

Dr. J. Smith Dodge, Jr. Would it not be the case usually that where most of us would speak of a "large" filling, you would count it two, three, or four, because, originally, it began with so many points?

Dr. Brockway. Certainly.

Dr. N. W. Kingsley. You charge for four fillings in that case?

Dr. Brockway. I do not, as a general thing.

Dr. N. W. Kingsley. This suggests a topic which I would like to dwell upon for a moment. I once had an unfortunate lawsuit where the question of charges was brought up. Several professional brethren were called by my opponents to prove that my charge of ten dollars an hour for filling teeth with gold was very unjust. They insisted that they did not charge by the hour; it was not a proper thing to do. On cross-examination it appeared, however, that the results of their method of charging did not bring them less than twenty dollars an hour.

The matter of charges has really been a question which I have thought upon considerably for many years, as to what is just to ourselves and our patients. While I make no rule for anybody else, I do say that I would not like to be charged by the operation, and have work done to the extent of filling a cavity every fifteen minutes, at the rate which it is now regarded among reputable dentists a man shall be paid for a filling properly inserted. When I am at work, I think my time ought to bring me so much money; and if I can put in four little fillings in an hour, my patient is entitled to the benefit of it rather than myself; and if it takes me two hours of faithful work to put in a filling only twice as big as one of the little ones, I am entitled to payment for two hours.

Dr. A. H. Brockway. I do not think it is any of the patient's business how long or how quickly the operation is done, so that it is done well. I have reason to suppose my patients are as well satisfied with an operation of fifteen minutes as if I took an hour to do the same thing; in fact, better satisfied. A gentleman who has been in my hands for ten or twelve years, and for whom I did a number of operations five or six years ago, came to me last summer, and I filled two cavities. The operation was very brief. He said to me, "You are not charging me any more than you used to." I said, "No; I don't think I charge quite as much." "Well," said he, "you have done this operation so much quicker than formerly, that I think you ought to charge more. As a business man, I am better satisfied to pay more for the same operation, because you have taken so much less time." I think that view of it will be taken by almost all business men.

Dr. Dwinelle. It seems to rest on this: Is it well done? And I must repeat that, as a rule, rapid operations cannot be done thoroughly.

Dr. Dodge. Is it a necessity that slow ones should be done better?

Dr. Dwinelle. No; but the rule must still stand good.

Dr. Mm. Jarvie, Jr. I do not know that Dr. Brockway requires any one to defend him. Still, I desire to say a word or two. In the first place, I think there is more or less misapprehension in the minds of some of the gentlemen present, certainly in the mind of Dr. Dwinelle, in regard to Dr. Brockway's paper. It did not state that these operations were all gold, and in a conversation with him I learned that perhaps not one-half of them were gold, the remainder being of such material that they required much less time than operations with gold would. The fillings of all descriptions that he put in within a period of eighteen days averaged fifteen minutes. Dr. Dwinelle has intimated rather strongly that these operations necessarily cannot be thorough. I have recently had opportunity to see quite a number of Dr. Brockway's operations, and I will vouch for the quality of them. In Brooklyn, where he is best known he is considered one of our most able and thorough operators. Of course, as to the record of time, we are to take his word for it. It would seem to some very singular that he should be able to adjust the rubber dam in such a short time. Sixty seconds is his average time per cavity for adjusting the rubber dam. Now, perhaps the fifteen minutes per operation would be no more marvelous, if you saw it done, than the adjustment of the rubber dam in the space of sixty seconds per filling.

Dr. C. P. Fitch. This matter of rapidity in operations is relative. When I entered the profession, one great object in operating was to see how rapidly fillings could be put in. It was so all through this state. There is a constitutional difference in men : some are phlegmatic, and so slow in movement that they cannot insert an ordinary filling in an hour ; others are mercurial, they are quick and nervous, and will fill a tooth just as well in fifteen minutes. I hold that a man should work to a standard. The patient has much to do with results obtained. I aim at a given result, whether it takes fifteen minutes or thirty, or a number of hours. I have worked at one tooth eleven hours, restoring and building up a gold crown.

In reference to the principle of rapidity of motion defeating itself. In the use of the engine, burs lose their bite from the fact that they clog. If burs fill up with *debris*, and do not cut until they are cleaned, we are not to suppose that they fail to cut because of the rapidity of the motion.

The engine, without proper manipulation, of course, is a detriment, and should be held as such. But I can do much more with it than I can with the hand instrument. If we had burs that would free themselves from the *debris*, we should have with rapidity of motion increase of power and and increase of result.

Dr. E. A. Bogue. I have seen patients of certainly three of the gentlemen who have just spoken within a few days, in my office. I saw one patient from Dr. Brockway some little time ago, and I confess, after seeing this patient, I have had an earnest desire to know how the doctor does his work. I have stood by and seen gentlemen operate, taking time enough, inflicting pain enough, and accomplishing slobber enough, for one whole day, over trifling crown cavities that could have been filled with perfect ease without the rubber dam. I have seen that sort of thing, and heard of it, until I am tired of it, and I was extremely glad to hear Dr. Brockway's paper on that point, simply as a matter of suggestion. As soon as I am able I want to go to Brooklyn and see how he does it, and then I can judge whether it is worth my while to strive to emulate his example. The patient that I did see I must say had most admirable fillings in his mouth, and if I am any judge, with the aid of some points that are rather searching under the magnifying-glass, I think those fillings will stay where they were put. I do not much think, however, that he put those fillings in in fifteen minutes, or an hour.

I am glad the question has been brought up and made as prominent as it has been. There is a deal of truth in Dr. Hill's criticism, a few meetings ago, that too much time is put upon much of our work. I do not mean to deprecate the most careful, nay, the most artistic work that we can do, where such work is called for; but I mean to discriminate between a restoration filling and in a tooth of such poor structure that it must necessarily break down inside of twelve months and a filling in a compact tooth that at the age of forty for the first time needs a restoration. I want to protest against the utter and sweeping condemnation pronounced by one of our professional gentlemen within a week or two upon all contour fillings, because he had seen a number of unsuccessful ones made in the mouth of a sixteen-year-old patient.

Dr. W. H. Dwinelle. I feel constrained to say a word or two further, to disclaim all thought of personality in anything I have said to-night. I respect a man none the less because he honestly differs with me; certainly this could never be ground for entertaining personal feelings.

Adjourned.—*Dental Cosmos.*

